# Upregulation of miR‐374a promotes tumor metastasis and progression by downregulating LACTB and predicts unfavorable prognosis in breast cancer

**DOI:** 10.1002/cam4.1576

**Published:** 2018-05-23

**Authors:** Jun Zhang, Yuting He, Yan Yu, Xiaolong Chen, Guangying Cui, Weiwei Wang, Xiaojian Zhang, Yonggang Luo, Juan Li, Fang Ren, Zhigang Ren, Ranran Sun

**Affiliations:** ^1^ Department of Pharmacy The First Affiliated Hospital of Zhengzhou University Zhengzhou China; ^2^ Precision Medicine Center The First Affiliated Hospital of Zhengzhou University Zhengzhou China; ^3^ Department of Pathology The First Affiliated Hospital of Zhengzhou University Zhengzhou China; ^4^ Key Laboratory of Clinical Medicine The First Affiliated Hospital of Zhengzhou University Zhengzhou China

**Keywords:** breast cancer, LACTB, metastasis, miR‐374a, proliferation

## Abstract

Breast cancer (BRCA) is the second leading cause of cancer‐related death among female worldwide. Recent studies have revealed that LACTB was frequently repressed and functioned as a bona fide new tumor suppressor in a series of cancers, including BRCA. However, the molecular mechanisms underlying LACTB dysregulation in BRCA have not been reported. In the present study, we find that LACTB is repressed in BRCA and associated with poor prognosis by BRCA tissue microarray (TMA) analysis. Moreover, we confirm that LACTB is a direct target of miR‐374a, which is significantly overexpressed and associated with malignancies in BRCA. Mechanistically, applying loss‐of‐function and gain‐of‐function approaches in a series of in vitro and in vivo experiments show that miR‐374a knockdown suppresses the cell proliferative and colony formation activity, as well as migration and invasion capacity, but LACTB silencing in these cells reverses this change. Furthermore, we find that miR‐374a silencing markedly reduces the tumor growth in xenograft mouse models. In summary, our findings suggest the miR‐374a/LACTB axis plays a critical role in the tumorigenicity and progression of BRCA. miR‐374a/LACTB axis may be a potential target in the development of therapeutic strategies for BRCA patients.

## 1 BACKGROUND

Globally, breast cancer (BRCA) is the second leading cause of cancer‐related death in women all over the world.^1^ In the United States, it is estimated that 252 710 new cases and 40 610 deaths occur from breast cancer during 2017.^2^ In spite of many improvements have been made in early diagnosis and more effective therapeutic strategies, tumor recurrence and metastasis are still inevitable in most patients diagnosed with early‐stage BRCA.^3^ In addition, resistance to chemotherapy is also a significant obstacle for BRCA treatments. BRCA is a highly heterogeneous disease and distinct gene expression profiles are related to pathologic processes and outcome in different BRCA subtypes.^4^ Thus, it is extremely urgent to unravel the molecular mechanisms underlying BRCA tumorigenesis and progression. Furthermore, exploring novel biomarkers and therapeutic targets is of outmost importance to improve breast cancer prognosis.

Recently, the mitochondrial gene beta‐lactamases (LACTB) had been reported to be frequently downregulated and involved in the progression of BRCA. As a mammalian active‐site serine protein, LACTB has evolved from a bacterial penicillin‐binding protein.^5^ Mechanism studies revealed that LACTB functioned as a tumor suppressor through regulating lipid metabolism and differentiation of BRCA cells.^6^ LACTB overexpression could significantly decrease the proliferation of BRCA cells. In addition, LACTB was substantially downregulated and correlated with a poor outcome of glioma patients.^7^ Overexpression of LACTB could inhibit the proliferation, invasion, and angiogenesis of glioma cells by regulating expression of PCNA, MMP2, MMP9, and VEGF.^7^ All these results indicated LACTB was a bona fide new tumor suppressor.^6^, ^8^ However, the relationship between LACTB expression and prognosis in BRCA, as well as the regulatory network of LACTB remain largely unclear.

MicroRNAs (miRNAs) represent a class of small non‐coding RNAs that bind to the 3′UTR region of target genes and inhibit their expressions by either promoting mRNA degrading or repressing their translation.^9^ A growing body of evidences have revealed a critical role of miRNAs in the regulation of numerous cellular functions.^10^ Additionally, emerging evidence have indicated that large number of miRNAs were frequently deregulated and involved in cancer development and progression, including BRCA.^11^, ^12^ Recent study demonstrated for the first time that miR‐125b‐5p was involved in atherosclerosis by directly targeting LACTB in THP‐1 macrophages.^13^ Therefore, we speculated miRNA might contribute to the dysregulation of LACTB in BRCA. Although LACTB has a critical role in BRCA, the interaction between LACTB and miRNAs remains unknown.

In this study, we demonstrated that LACTB was frequently dysregulated in cancers. Moreover, downregulated LACTB was significantly associated with poor clinical prognosis of breast cancer by TMA analysis. Furthermore, using bioinformatics prediction and dual luciferase reporter, we confirmed that LACTB was the direct target of miR‐374a, which was observably overexpressed in BRCA tissues. Functionally, miR‐374a knockdown significantly inhibited proliferation and invasion of BRCA cells in vitro and tumor growth in vivo by upregulating LACTB expression. Collectively, our findings indicate that miR‐374a/LACTB axis contributes to the progression of BRCA, suggesting that miR‐374a/LACTB axis can be considered a potential prognostic marker and therapeutic target for BRCA patients.

## 2 METHODS

### 2.1 Patient samples and tissue microarray

A Pan‐cancer Tissue microarray (Pan‐cancer TMA) containing 10 common types of cancers (each type cancer containing 20 specimens and matched adjacent non‐tumor tissues) were constructed with clinical specimens acquired between April 2016 and December 2016 from the First Affiliated Hospital of Zhengzhou University, Zhengzhou University, China. TMA containing 127 BRCA specimens (Outdo cohort) was purchased from Outdo Biotech (Shanghai, China). The clinicopathological data for these patients are presented in Table S1. The study was approved by the Institutional Review Board of the First Affiliated Hospital of Zhengzhou University. All patients provided written informed consent and the project was in accordance with the Helsinki Declaration of 1975.

### 2.2 Cell lines and cell culture

Human breast cancer cell lines (MDA‐MB‐231 and MCF‐7) were obtained from the American Type Culture Collection (Rockville, MD) and incubated in a CO_2_ incubator (5% CO_2_/95% air) at 37°C in DMEM medium (Gibco, USA) with 10% fetal bovine serum (CLARK Bioscience, USA). All cell lines used in this study had been passed for less than 4 months in culture when the experiments were carried out.

### 2.3 PCR assays and western blotting analysis

Total RNA was extracted from cells with Trizol reagent (TAKARA, Japan) following the manufacturer’s protocol. qRT‐PCR was performed with 7500 fast (Applied Biosystems, USA) after all mRNAs and miRNAs were reverse transcribed according to the product description of the PrimeScript RT Master Mix Perfect Real Time (TAKARA, Japan). The primer sequences used in this study are listed in Table S1.

The procedure of western blotting was performed as described previously.^14^ Briefly, proteins were extracted from cultured cells using RIPA protein extraction reagent (Beyotime, China), then separated by 12% SDS‐PAGE gels and transferred to polyvinylidene difluoride (PVDF) membranes (Millipore, USA). After blocked with PBS containing 5% skim milk, the membranes were treated with the primary antibodies at 4°C overnight. The membranes were incubated with secondary antibodies after washing with TBST 3 times. Membrane signals were scanned using the Odyssey infrared imaging system and analyzed using Odyssey 3.0 software (LI‐COR Biosciences). The antibodies used in this study are listed in Table S3.

### 2.4 Transfection of cell lines

miR‐374a inhibitors were commercially purchased from GenePharma (Shanghai, China). Breast cancer cell lines were transfected with miR‐374a inhibitors or LACTB siRNA using Lipofectamine 3000 transfection reagent (Invitrogen, USA), according to the manufacturer’s instructions. The sequences of the miR‐374a inhibitors and negative control are listed in Table S2.

### 2.5 TCGA datasets analysis

To analyze the expression levels of miR‐374a and LACTB mRNA in cancers, we downloaded and analyzed mRNA/miRNA gene expression datasets and clinical data from the Cancer Genome Atlas Project (TCGA; http://tcga-data.nci.nih.gov/) for BRCA patients including a total of 534 BRCA tumor samples.

### 2.6 Luciferase activity assay

Both wild‐type and mutant 3′UTR region of the LACTB mRNA were subcloned into psiCHECK‐2 vector (Promega, USA). Cells were co‐transfected with 200 ng of wild‐type or mutated vectors and 80 nmol/L miR‐374a. After 48 hours, luciferase enzyme activity, normalized to firefly luciferase activity, was determined with the Dual‐Luciferase Reporter Assay System (Promega, USA).

### 2.7 Cell growth assay

MTT assays were performed as follows. BRCA cells were transfected with miRNA or si‐RNA. At 1, 2, 3, 4, and 5 days after incubation, proliferation was assessed using MTT (3‐(4,5‐dimethylthiazol‐2‐yl)‐2,5‐diphenyltetrazolium bromide) solution (Beyotime, China). The formazan crystals were dissolved with DMSO, and absorbance was read by spectrophotometer (Molecular Devices, USA).

For the colony formation assay, 1000 cells were placed into six‐well plate at 24 hours after transfection. Incubated for 14 days in CO_2_ incubator, the cells were fixed and dyed with 0.1% crystal violet. The colonies (defined as over than 50 cells) number was counted. The DNA synthesized rate was determined using EdU assay kit (Ribobio, Guangzhou, China) according to the product description.

### 2.8 Invasion and wound healing assay

Cell invasion assay was performed with BD BioCoat Matrigel invasion chambers (BD, USA) following the manufacturer’s protocol. The procedure was performed as previously described. Wound healing assay was carried out to determine the cell migration. In brief, the cells were plated in six‐well plate and incubated. When cells reached 80%‐90% confluence, we created a gap by scratching a confluent monolayer with a 200 μL pipette tip. The wounds were observed at 0, 24, and 36 hours with microscope.

### 2.9 Immunohistochemical (IHC) staining

IHC was executed as previously described.^14^ Briefly, sample sections were deparaffinized through xylene and rehydrated with graded alcohol, antigen retrieved by citrate buffer, blocked with bovine serum albumin, and then incubated with the primary antibodies overnight. Subsequently, the sections were incubated with secondary antibodies at room temperature, and the nuclei were counterstained with hematoxylin. The images of IHC and H&E staining were obtained using the NanoZoomer 2.0‐RS system (Hamamatsu Photonics Inc., Germany), and the digital slides were analyzed by the software NDP.view 2.5.14 version.

### 2.10 In vivo tumor growth experiments

The athymic BALB/C mice (4‐ to 6‐weeks old) were purchased from Beijing Vital River Laboratory Animal Technology (Beijing, China). MCF‐7 cells (2 × 10^6^) transfected with miR‐374a (sh‐miR‐374a) or negative control shRNA (NC) were injected subcutaneously into the nude mice. Tumor growth was monitored with tumor weight, and photographed by IVIS@ Lumina II system (Caliper Life Sciences, Hopkinton, MA). All the in vivo experiments were approved by the institutional animal care and use committee of the First Affiliated Hospital of Zhengzhou University.

### 2.11 Statistical analysis

The statistical analyses were performed using GraphPad Prism software (version 6.0, GraphPad Software, Inc., La Jolla, CA) or the SPSS software (version 23.0, SPSS Inc., Chicago, IL). The significance of the correlation of LACTB expression with clinicopathological characteristics in BRCA was investigated by the chi‐squared test. Mann‐Whitney *U* test and unpaired *t* test were used to analyze the continuous variables. Pearson’s correlation was performed to assess the linear association between 2 variables. Survival analysis was performed with available survival data from TMA using log‐rank test. A two‐sided statistical significance level of 0.05 was used for all statistical analyses.

## 3 RESULTS

### 3.1 LACTB is frequently dysregulated expression in cancers

**Figure 1:**
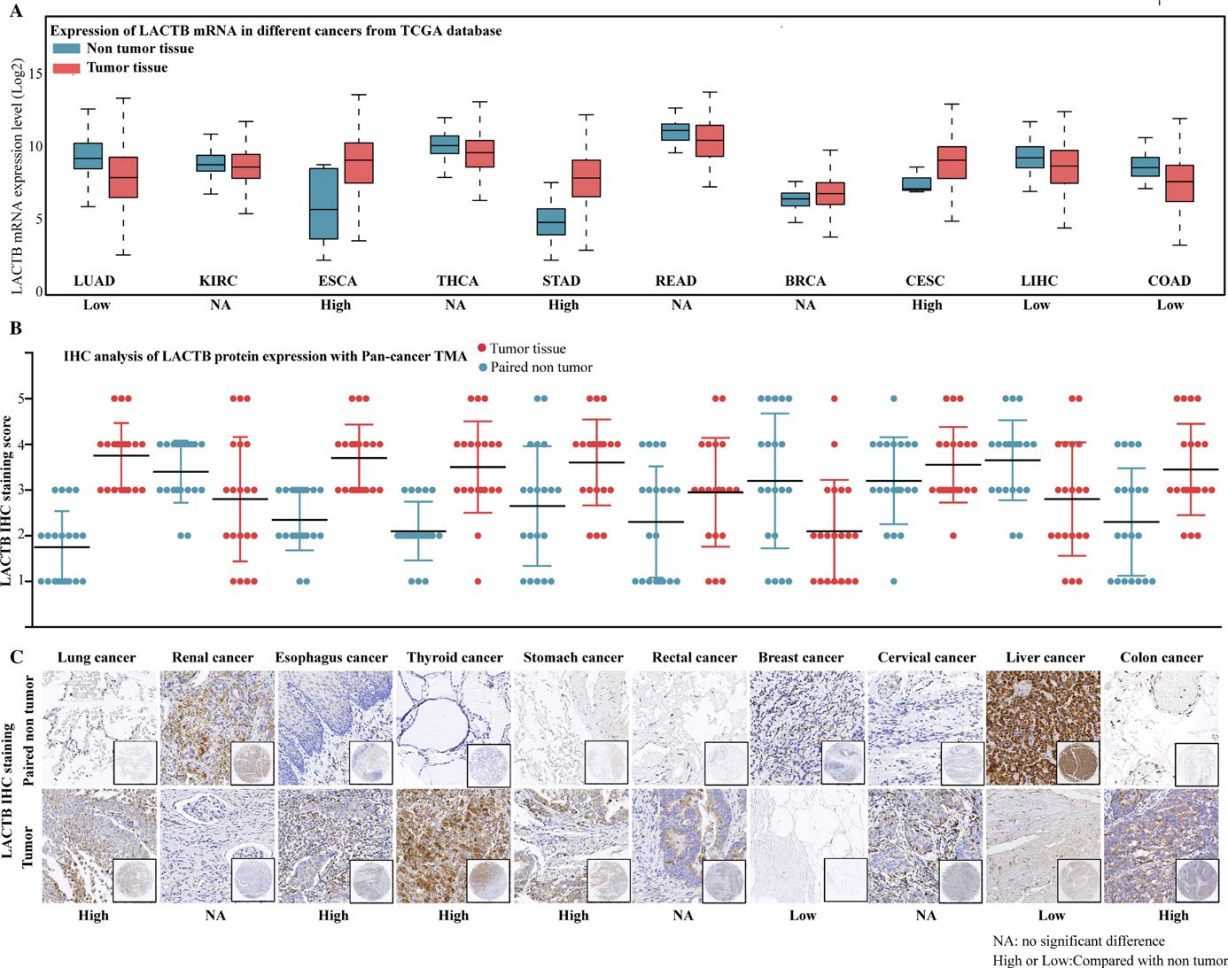
LACTB is frequently dysregulated expression in cancers. A, LACTB mRNA expression level in TCGA data analysis. LACTB mRNA was upregulated in tumor tissues of esophagus cancer (ESCA), stomach cancer (STAD), and cervical cancer (CESC), whereas low downregulated in lung cancer (LUAD), liver cancer (LIHC), and colon cancer (COAD), there was no significant difference in breast cancer (BRCA), rectal cancer (READ), thyroid cancer (THCA), and renal cancer (KIRC). B, LACTB protein expression in pan‐cancer tissues and paired non‐tumor samples. LACTB protein was upregulated in tumor tissues of lung cancer, esophagus cancer, thyroid cancer, stomach cancer, and colon cancer, whereas low downregulated in liver cancer and breast cancer, there was no significant difference in renal cancer, rectal cancer, and cervical cancer. C, Representative LACTB histologic scoring in pan‐cancer tissues and paired non‐tumor samples

To determine the expression of LACTB in most common cancers, TCGA data analysis was performed to identify LACTB mRNA expression level. As shown in Figure 1A, LACTB mRNA expression was frequently dysregulated in cancers, such as upregulation in tumor tissues of ESCA, STAD, and CESC, whereas downregulation in tumor tissues of LUAD, LIHC, and COAD, compared with normal tissues. However, there was no significant difference in BRCA, inconsistent with the reported low‐expressed LACTB in BRCA.^6^ This contradictory result might be due to the complicated regulatory mechanism of post‐transcriptional control. In view of the expression difference in mRNA and protein, we further analyzed the expression difference of LACTB protein in Pan‐cancer TMA. A low expression of LACTB protein was observed in BRCA tissues compared with matched adjacent non‐tumor tissues by Pan‐cancer TMA analysis, as well as in liver cancer (Figure 1B,C). These results confirmed the downregulated LACTB protein in BRCA and indicated the tumor‐specific expression pattern of LACTB, which indicated LACTB may perform different functions in different tumors.

### 3.2 Low expression of LACTB was correlated with prognosis of BRCA patients

**Figure 2:**
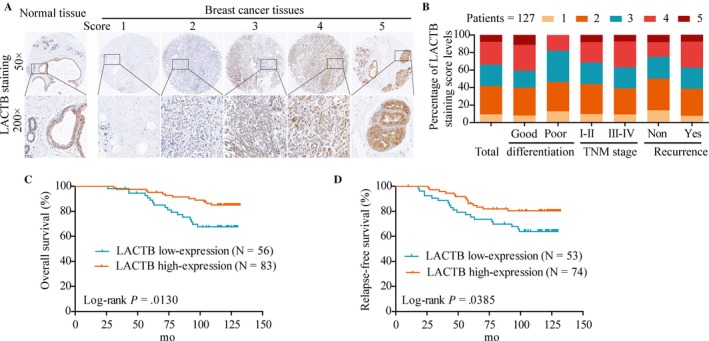
Low expression of LACTB was correlated with prognosis of breast cancer patients. A, Representative LACTB and immunohistochemical staining patterns with different staining scores in BRCA tissues. B, Distribution of LACTB immunohistochemical staining scores in BRCA tissues according to TNM classification, with or without recurrence. C, Kaplan‐Meier overall survival analysis between expression of LACTB (red, high LACTB expression; blue, low LACTB expression). D, Kaplan‐Meier relapse‐free survival analysis between expression of LACTB (red, high LACTB expression; blue, low LACTB expression)

To determine the clinical significance of low LACTB expression in BRCA, the LACTB expression was analyzed in BRCA TMA by IHC. According to staining intensity, LACTB staining was scored from 1+ to 5+ and the score distribution in patients with different clinical features (Figure 2A,B). Scores 1+ to 3+ were defined as LACTB low expression, whereas scores 4+ to 5+ as LACTB high expression. As shown in Figure 2C,D, Kaplan‐Meier analysis revealed that low expression of LACTB was significantly associated with poor overall survival and relapse‐free survival in BRCA patients. Moreover, univariate Cox proportional hazards analysis exhibited that LACTB expression, distant metastasis, pathologic grade, and TNM stage were important prognostic factors, and multivariate Cox regression analysis showed that low expression of LACTB was an independent prognostic factor for predicting poor survival of BRCA (Tables 1 and 2). Collectively, these findings strongly suggested that downregulated LACTB was positively associated with poor prognosis and might serve as a prognostic biomarker and therapeutic target in BRCA.

**Table 1:** Univariate and multivariate analyses of overall survival of breast carcinoma

	Clinicopathological features	Univariate analyses			Multivariate analyses		
		HR	95% (CI)	*P* value	HR	95% (CI)	*P* value
Age (y)	≤Median	1.000		.004**	1.000		.001**
	>Median	1.050	1.016‐1.086		1.063	1.025‐1.102	
Her‐2	Positive	1.000		.024*	1.000		.022*
	Negative	2.622	1.138‐6.038		2.821	1.160‐6.859	
Pathologic pattern	Invasive ductal carcinoma	1.000		.512			
	Others	1.385	0.522‐3.674				
TNM stage	Stage I and II	1.000		.001**	1.000		.000**
	Stage III and IV	4.206	1.767‐10.014		5.172	2.105‐12.708	
Differentiation grade	Stage I‐II	1.000		.040*	1.000		.106
	Stage III‐IV	2.247	1.039‐4.861		1.955	0.868‐4.406	
LACTB expression	Low	1.000		.028*	1.000		.018*
	High	0.684	0.488‐0.959		0.640	0.443‐0.925	

CI, confidential interval; HR, hazard ratio; LACTB, Lactamase Beta; TNM, tumor node metastasis.

*P <* *.05, **P < .001.

**Table 2:** Univariate and multivariate analyses of relapse‐free survival of breast carcinoma

	Clinicopathological features	Univariate analyses			Multivariate analyses		
		HR	95% (CI)	*P* value	HR	95% (CI)	*P* value
Age (y)	≤Median	1.000		.013*	1.000		.003**
	>Median	1.042	1.009‐1.076		1.052	1.017‐1.089	
Her‐2	Positive	1.000		.016*	1.000		.017*
	Negative	2.778	1.205‐6.403		2.900	1.214‐6.927	
Pathologic pattern	Invasive ductal carcinoma	1.000		.384			
	Others	1.543	0.582‐4.092				
TNM stage	Stage I and II	1.000		.001**	1.000		.001**
	Stage III and IV	4.310	1.810‐10.263		4.410	1.839‐10.578	
Differentiation grade	Stage I‐II	1.000		.030*	1.000		.026*
	Stage III‐IV	2.354	1.087‐5.094		2.529	1.116‐5.730	
LACTB expression	Low	1.000		.021*	1.000		.029*
	High	0.677	0.477‐0.960		0.620	0.417‐0.921	

CI, confidential interval; HR, hazard ratio; LACTB, Lactamase Beta; TNM, tumor‐node‐metastasis.

*P <* *.05, **P < .001.

### 3.3 LACTB is the functional target of miR‐374a

**Figure 3:**
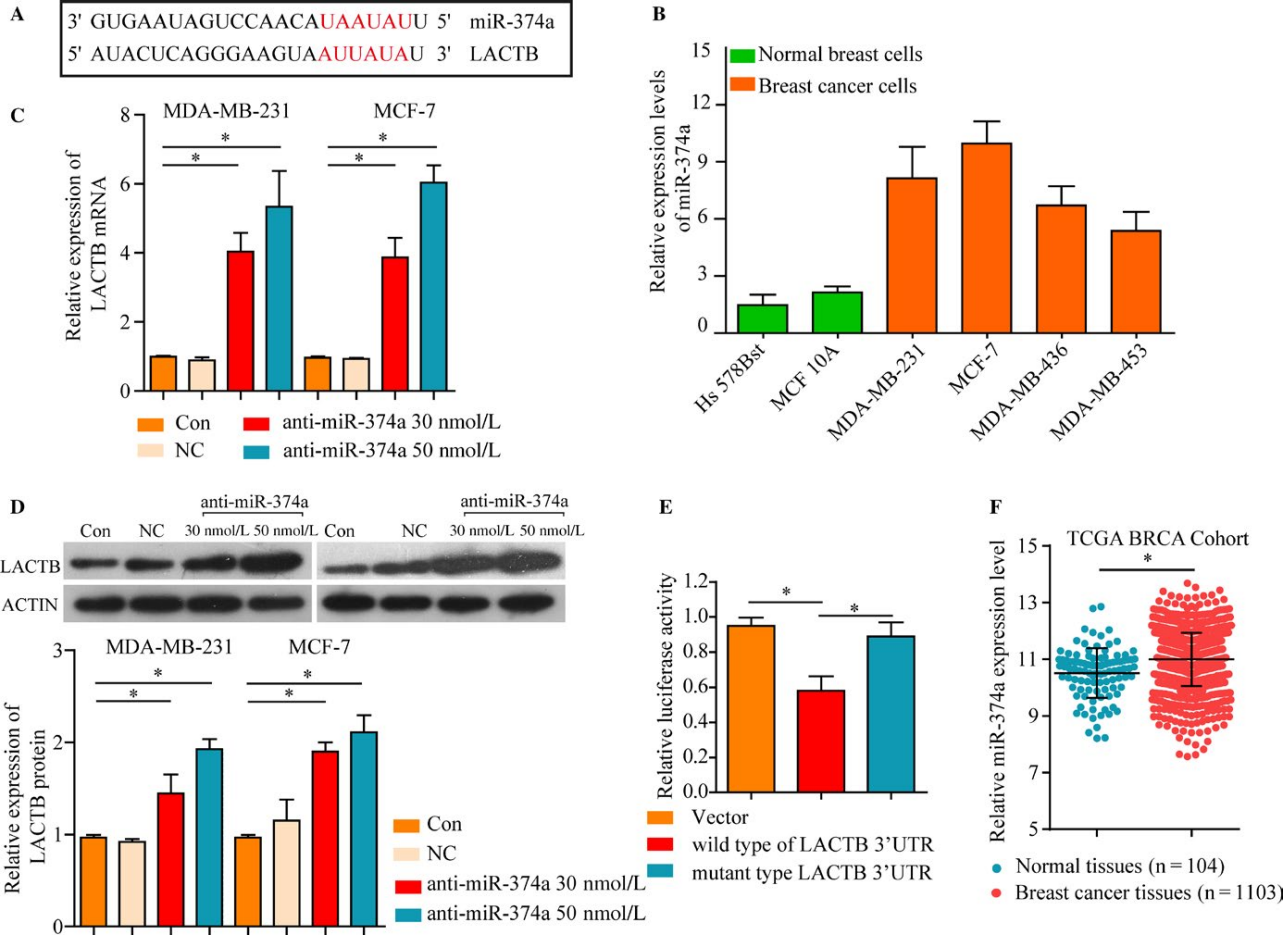
LACTB as the functional target of miR‐374a. A, Schematic representation of the miR‐374a site in LACTB 3′‐UTR. B, miR‐374a expression was significantly higher in MDA‐MB‐231 and MCF‐7 compared with MDA‐MB‐436, MDA‐MB‐453 breast cancer cells and Hs578Bst, MCF 10A normal breast cells. The effect of miR‐374a inhibitors or NC on the expression of LACTB in MDA‐MB‐231 and MCF‐7 cells was determined by (C) mRNA and (D) western blot. E, Luciferase assays using LACTB 3′UTR region with either wide‐type miR‐374a binding site or mutant binding site after ectopic expression of miR‐374a. F, miR‐374a expression levels in TCGA BRCA cohort. *P < .05

In light of the critical role of LACTB and mechanism of action of miRNAs, we speculated miRNA might contribute to the dysregulation of LACTB. Bioinformatics online databases (Targetscan, Miranda) were used to screen miRNAs which have potential binding site for the 3′‐UTR of LACTB. According to the comprehensive analysis results, miR‐374a, as shown in Figure 3A, was chosen for further validation. First, compared with normal breast cell lines, we determined notably high expression of miR‐374a in BRCA cell lines, especially in MDA‐MB‐231 and MCF‐7 cells (Figure 3B). Therefore, we transfected miR‐374a inhibitors into MDA‐MB‐231 and MCF‐7 cells for further study. Then, qPCR was performed to detect LACTB mRNA expression in transfected cells and results showed that LACTB was upregulated in MDA‐MB‐231 and MCF‐7 cells treated with miR‐374a inhibitor (Figure 3C). Subsequently, we found that miR‐374a silencing obviously increased LACTB protein expression in BRCA cells (Figure 3D). Furthermore, luciferase reporter assay showed that luciferase activity was remarkably decreased in wild LACTB luciferase reporter plasmid transfected cells, compared to the mutant‐type LACTB or negative control group, suggesting the specificity of the interaction between miR‐374a and LACTB (Figure 3E). Additionally, we analyzed a TCGA BRCA dataset to identify the expression of miR‐374a in BRCA, and confirmed that miR‐374a expression level was statistically significantly higher in BRCA tissues than that in normal adjacent tissues (Figure 3F). Collectively, these findings suggested that upregulated miR‐374a could directly target the 3′UTR of LACTB in BRCA.

### 3.4 miR‐374a expression is critical for BRCA cell growth and survival

**Figure 4:**
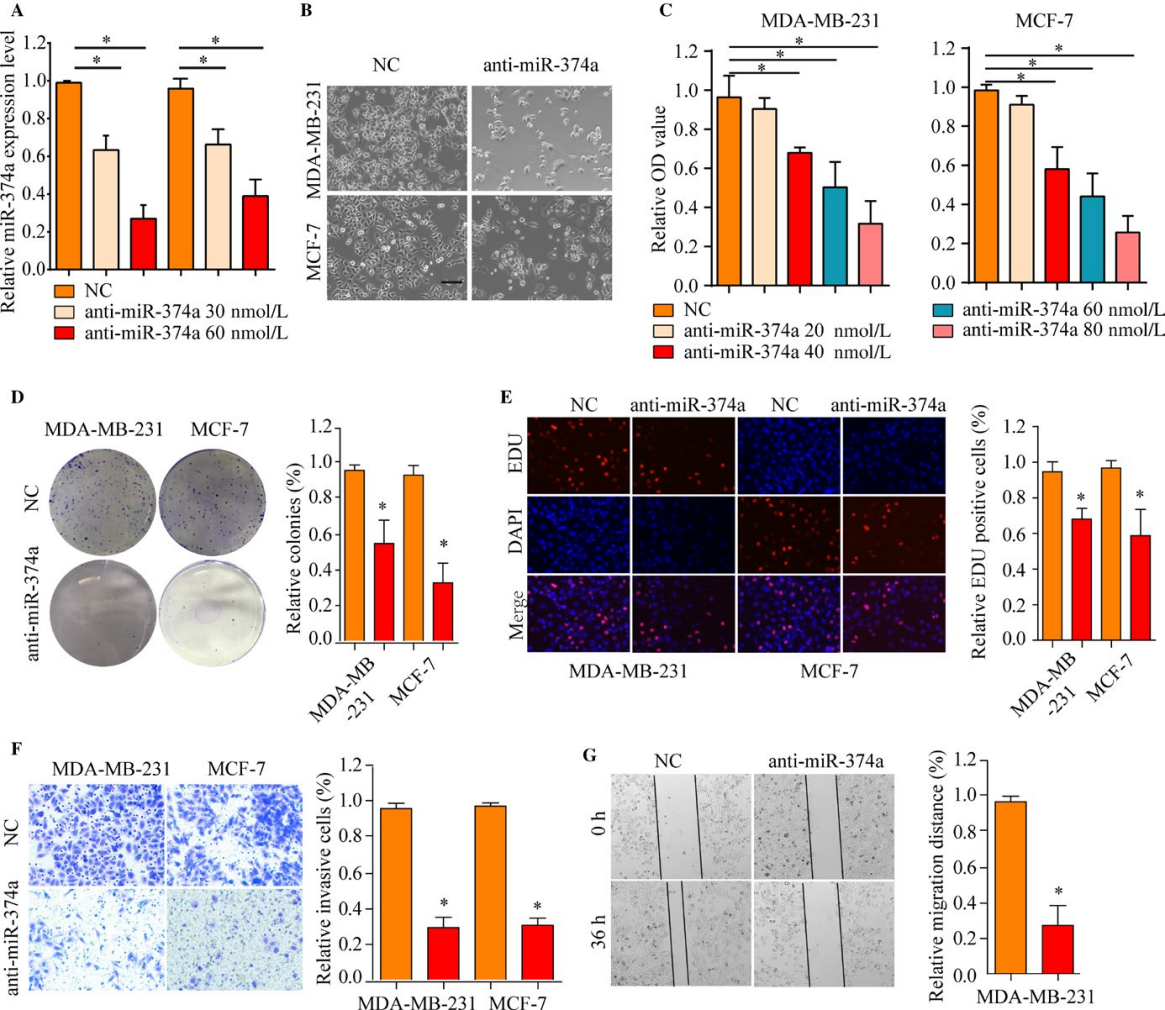
Inhibiting miR‐374a suppresses BRCA proliferation and invasion. A, miR‐374a mRNA expression was decreased after transfection with miR‐374a inhibitor. B, miR‐374a silencing attenuated proliferation of MDA‐MB‐231 and MCF‐7 cells. C, Dose‐dependent anti‐miR‐374a downregulated the expression of miR‐374a. D, The number of colonies was considerably lower for cells transfected with anti‐miR‐374a compared to respective controls. E, EdU assay showed that treated with miR‐374a inhibitor could suppress proliferation of MDA‐MB‐231 and MCF‐7 cells. F, The invasiveness of MDA‐MB‐231 and MCF‐7 cells infected with miR‐374a inhibitor was significantly suppressed according to cell invasion assay. G, miR‐374a silencing caused a remarkable suppression of cell migration in MDA‐MB‐231 cells using wound‐healing assay. *P < .05

The above observations prompted us to explore the potential biologic function of miR‐374a in BRCA tumorigenesis and progression. Initially, decreased expression of miR‐374a after transfection with miR‐374a inhibitor was confirmed by real‐time PCR (Figure 4A). Proliferation assays indicated that downregulation of miR‐374a inhibited the proliferation capacity of MDA‐MB‐231 and MCF‐7 cells (Figure 4B,C). Consistently, the number of colonies was lower for cells transfected with miR‐374a mimics compared to respective negative controls (Figure 4D). In addition, EdU assays manifested that compared with negative control group, miR‐374a knockdown significantly inhibited proliferative activity of BRCA cells (Figure 4E).

Next, the effect of miR‐374a on migration and invasion capacity of BRCA cells was characterized. Transwell invasion assays indicated a reduced invasiveness of BRCA cells (Figure 4F). Furthermore, miR‐374a silencing caused a remarkable suppression of cell migration in MDA‐MB‐231 and MCF‐7 cells using wound‐healing assay (Figure 4G). Thus, these data showed that miR‐374a plays an important role in regulating cellular events related to cancer proliferation, migration, and invasion.

### 3.5 miR‐374a’s oncogenic activity is in part through negative regulation of LACTB in vitro

**Figure 5:**
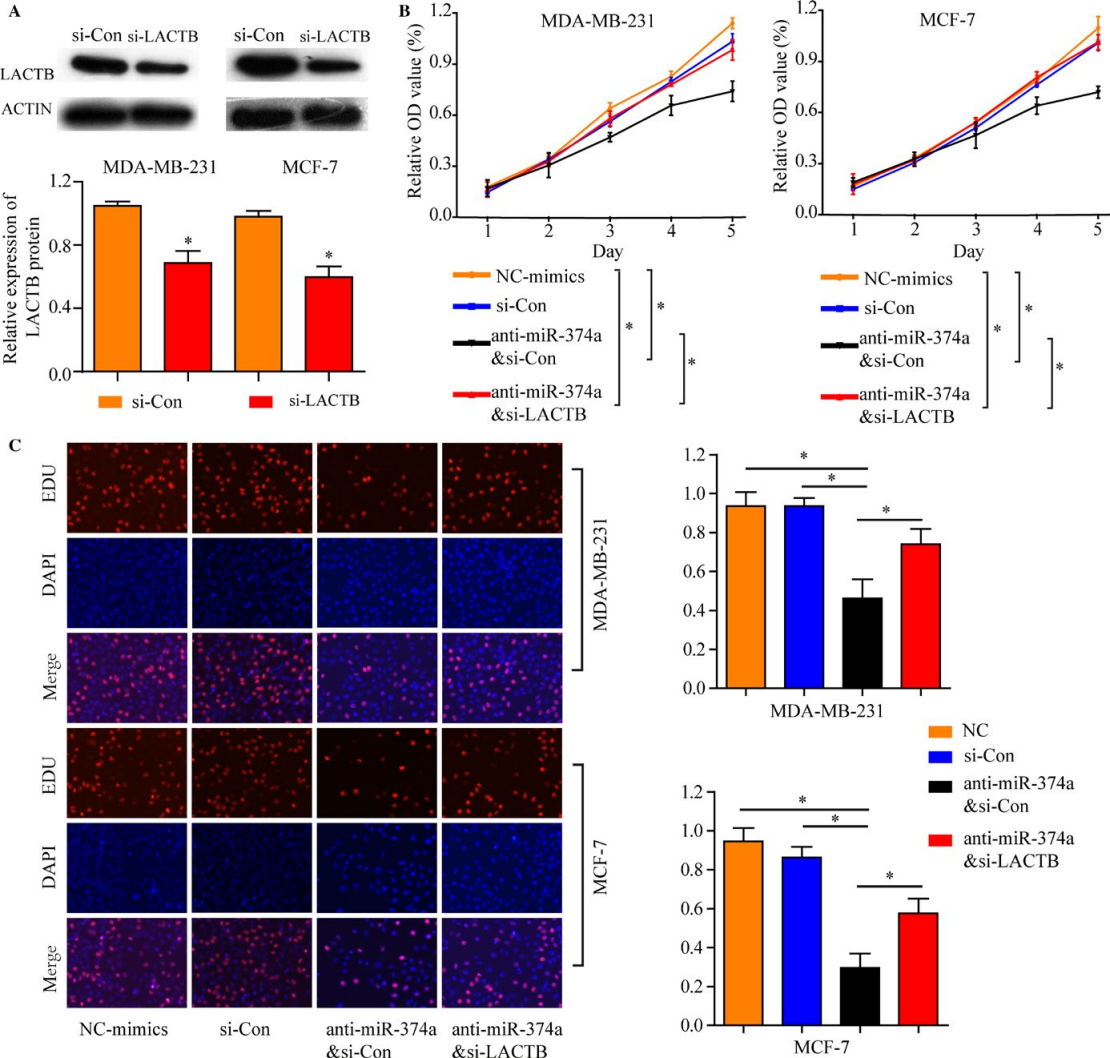
miR‐374a’s oncogenic activity is in part through negative regulation of LACTB in vitro. A, LACTB protein expression was decreased after transfected with siRNA targeting LACTB (LACTB‐siRNA) or siRNA negative control (NC) in MDA‐MB‐231 and MCF‐7 cells. B, Proliferation assays indicated that downregulation of miR‐374a inhibited the proliferation capacity of MDA‐MB‐231 and MCF‐7 cells, which was partially restored when LACTB was inhibited by RNA interference. C, EdU assays indicated that compared with negative control groups, miR‐374a knockdown significantly inhibited proliferative activity, which was significantly reversed by the LACTB silencing. *P <* *.05, **P < .01

To confirm whether miR‐374a promotes the BRCA cells proliferation, migration, and invasion by targeting LACTB, we performed loss‐of‐function and gain‐of‐function experiment in vitro. Initially, MDA‐MB‐231 and MCF‐7 cells were transfected with siRNA targeting LACTB (LACTB‐siRNA) or siRNA negative control (NC), and the results showed that LACTB protein expression was decreased in LACTB‐siRNA group (Figure 5A). Subsequently, proliferation assays indicated that downregulation of miR‐374a inhibited the proliferation capacity of MDA‐MB‐231 and MCF‐7 cells, which was partially restored when LACTB was inhibited by RNA interference (Figure 5B). Consistently, EdU assays certified that compared with negative control group, miR‐374a knockdown decreased proliferative activity, which was significantly reversed by the LACTB silencing (Figure 5C). Thus, these data confirmed that miR‐374a could function as an oncogene in BRCA, at least in part, by targeting LACTB.

### 3.6 The oncogenic activity of miR‐374a is in part through negative regulation of LACTB in vivo

**Figure 6:**
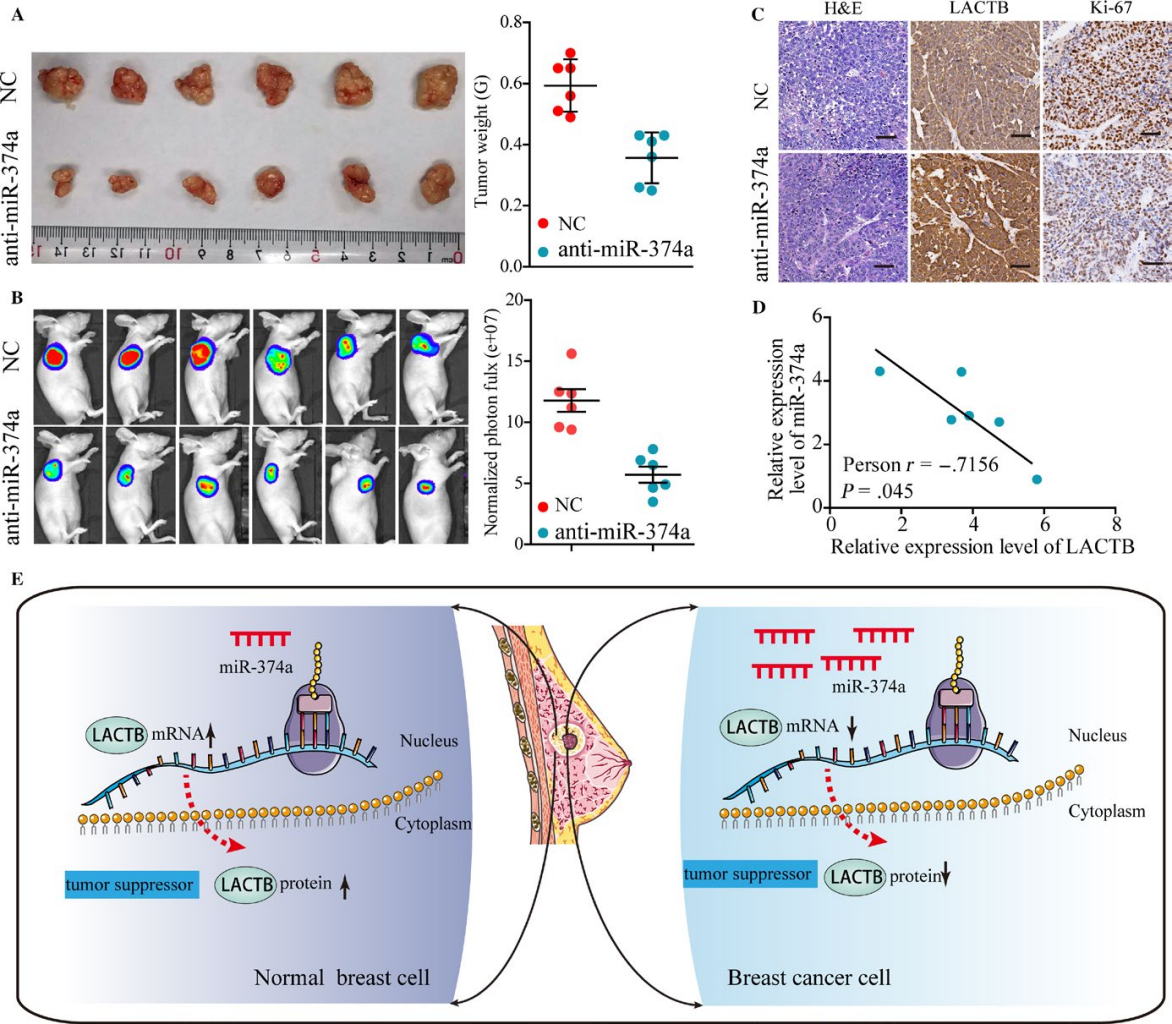
The inhibition of miR‐374a suppress BRCA tumorigenesis in vivo. MCF‐7 cells stable transfected with miR‐374a shRNA (lenti‐miR‐374a) or vector (Lenti‐mock) were injected into nude mice subcutaneously (n = 12). A, Tumor volume and tumor weight in lenti‐miR‐374a group were markedly smaller than those of Lenti‐mock group. B, Images of tumor formation were performed by a live imaging system detecting the luciferase signal. The luciferase activity of the lenti‐miR‐374a tumors was lower than that of the Lenti‐mock group. C, Sections of xenograft tumors stained with hematoxylin and eosin (H&E), as well as immunohistochemical staining for LACTB and Ki‐67. D, miR‐374a expression in relation to the expression levels of LACTB mRNA. E, Schematic representation showing that miR‐374a could function as a tumor oncogene and contribute to breast cancer development, at least in part, through targeting LACTB

To further elucidate that the oncogenic activity of miR‐374a is in part through the negative regulation of LACTB, lentivirus construct with the control or sh‐miR‐374a were stably transfected into MCF‐7 cells and then injected these cells subcutaneously into nude mice. Tumor volumes and luciferase signals were measured on a weekly basis. After 4 weeks, we observed that miR‐374a knockdown observably reduced the tumor volume and weight when compared with control group (Figure 6A). Consistently, luciferase signals in the sh‐miR‐374a group were signally decreased (Figure 6B). In addition, immunohistochemical staining for LACTB and Ki‐67 of xenograft tumors was performed. Stronger staining of LACTB and weaker Ki‐67 were observed in the tumor from the sh‐miR‐374a by IHC analysis (Figure 6C). Moreover, LACTB expression negatively correlated with miR‐374a expression in 12 xenograft tumors tissues as shown in Figure 6D. Taken together, these findings verified that miR‐374a promotes cancer progression by downregulating LACTB expression in BRCA.

## 4 DISCUSSION

LACTB, localized in the mitochondrial, is a mammalian active‐site serine protein and most prominently expressed in skeletal, heart, muscle, and liver. It has been shown that LACTB promotes intra‐mitochondrial membrane organization and regulates cellular metabolic processes. Increasing evidence indicated that LACTB was a novel protease homologues involved in energy homeostasis and could regulate the metabolic circuitry.^15^, ^16^, ^17^ In addition, dysregulated LACTB was associated with obese phenotype and atherosclerosis, which indicated the role of LACTB in fat metabolism disorder.^18^ Moreover, metabolic reprogramming is a cancer hallmark and abnormal fatty acid metabolism is related to the cancer progression, suggesting key enzymes of fatty acid metabolism may be served as a novel therapeutic target in cancers. Recent studies suggested that downregulated LACTB promoted cell proliferation in BRCA and glioma.^6^, ^7^

Through TCGA datasets and Pan‐cancer TMA analysis, we confirmed that both LACTB mRNA and protein were frequently dysregulated expression in cancers. A low expression of LACTB protein was observed in BRCA and liver cancer, indicating a tumor‐specific expression pattern of LACTB. Subsequently, we found that LACTB was low expressed in BRCA tissues and significantly associated with a shorter overall survival and relapse‐free survival time of BRCA patients. Additionally, Cox proportional hazard regression analysis further identified low expression of LACTB as an independent factor for poor outcome in BRCA patients. These data indicated that low LACTB expression was associated with aggressive behavior and an unfavorable prognosis in BRCA, consistent with the results observed in glioma.^7^ Thus, understanding the biologic basis for the observed deregulation of LACTB is of great value for future development of novel therapeutic strategies.

Emerging evidence indicated that miRNAs play a significant role in cancer pathogenesis by downregulating target genes. A growing body of evidence suggested that miR‐374a could function as a oncogene in various cancer progression, such as lung cancer,^19^ gastric cancer,^20^ ovarian cancer,^21^ liver cancer,^22^ osteosarcoma,^23^ and nasopharyngeal carcinoma.^24^ Additionally, miR‐374a was overexpressed in BRCA tissues from patients with distant metastases and was related with poor survival through activating Wnt/β‐catenin signaling.^25^ Consistently, in this study, we confirmed that miR‐374a was significantly upregulated in human BRCA, suggesting the oncogene role of miR‐374a in BRCA.

Thereafter, we performed a bioinformatics prediction for potential mRNAs targeted by miR‐374a and finally focused on LACTB due to its consentaneous function in tumorigenesis and progression. In addition, we carried out qPCR, western blot, and Luciferase reporter assays to validate the direct binding between miR‐374a and LACTB. Moreover, applying loss‐of‐function and gain‐of‐function approaches in a series of in vitro assays, we demonstrated that the miR‐374a/LACTB axis played a critical role in BRCA cell proliferation and invasion. Downregulation of miR‐374a significantly suppressed BRCA cell proliferation and colony formation activity, as well as migration and invasion capacity, whereas LACTB knockdown had the opposite effects. Consistent with the result in vitro, subcutaneous xenograft study showed that growth of xenografted tumor was dramatically suppressed in the sh‐miR‐374a group versus control group. Furthermore, results showed that there was remarkable difference in the LACTB staining between control and sh‐miR‐374a groups. This synergistically inhibitory effect in vivo strengthened the clinically significance of miR‐374a/LACTB axis in BRCA progression. Taken together, the above findings indicated that miR‐374a could function as a tumor oncogene and contribute to breast cancer development, at least in part, through targeting LACTB (Figure 6E).

Naturally, there are certain limitations in the present work. Given gene often be regulated by miRNAs, LncRNAs, or epigenetic processes and other mechanisms, other molecules might also contribute to the dysregulated of LACTB in BRCA. We did not determine whether miR‐374a/LACTB axis regulates fatty acid metabolism in BRCA cancer due to the limit of experimental instrument. Additionally, though miR‐374a/LACTB axis play a crucial role in BRCA, the potential clinical application of miR‐374a/LACTB axis remains a complex issue with big challenging.

## 5 CONCLUSION

Our findings demonstrate for the first time that miR‐374a contributes to tumorigenicity and progression in BRCA by suppressing LACTB expression, as confirmed using a series of in vitro and in vivo assays, and clinical patients’ samples. Our results also confirmed the vital role of the miR‐374a/LACTB axis in determining malignancy of BRCA. Thus, miR‐374a may be a novel prototype therapeutic agent that can target LACTB for BRCA patients.

## ACKNOWLEDGMENTS

Not applicable.

## CONFLICTS OF INTERESTS

The authors confirm that there are no conflicts of interest.

## AVAILABILITY OF DATA AND MATERIALS

Available under request.

## CONSENT FOR PUBLICATION

The participant gave informed consent before taking part in this study. All samples were de‐identified.

## ETHICS APPROVAL AND CONSENT TO PARTICIPATE

The study was approved by the human ethic committee of the First Affiliated Hospital of Zhengzhou University. All patients provided written informed consent and the project was in accordance with the Helsinki Declaration of 1975. Their clinical information would be kept in the databases of the First Affiliated Hospital of Zhengzhou University and utilized for research. All the in vivo experiments were approved by the institutional animal care and use committee of the First Affiliated Hospital of Zhengzhou University.
