# Supplementary material for: Upregulation of miR‐374a promotes tumor metastasis and progression by downregulating LACTB and predicts unfavorable prognosis in breast cancer
Source: Cancer Med. 2018 May 23;7(7):3351–62. doi: 10.1002/cam4.1576 (PMC6051141; doi:10.1002/cam4.1576)
Supplement: Supplementary file 1 [file CAM4-7-3351-s001.docx]

**Supplementary Table 1. qRT-PCR Primer sequence used in this study**

| **Name** | **sense sequence** | **anti-sense sequence** |
| --- | --- | --- |
| LACTB | 5’- CACCATGTACCG GCTCCTGTCAAG -3’ | 5’-TTAGTCAGCTCTGTCTTTATCAAATTC -3’ |
| β-actin | 5’-CTCCATCCTGGCCTCGCTGT -3’ | 5’-GCTGTCACCTTCACCGTTCC -3’ |
| miR-374a | 5’-ATTTTAGAGGAGGGGATT -3’ | 5’-TCACTTTAGCAGGCACAC -3’ |
| U6 | 5’-CTCGCTTCGGCAGCACA -3’ | 5’-AACGCTTCACGAATTTGCGT-3’ |

**Supplementary Table 2. siRNA sequence used in this study**

| **siRNA** | **sense sequence** |
| --- | --- |
| miR-374a inhibitors | 5’-CACUUAUCAGGUUGUAUUAUAA-3′ |
| Inhibitors negative control | 5’-CAGUACUUUUGUGUAGUACAA-3′ |

**Supplementary Table 3. Information on antibodies used in this study**

| **Antibody** | **WB** | **IHC** | **IF** | **Specificity** | **Company** |
| --- | --- | --- | --- | --- | --- |
| β-actin | 1:5000 | / | 1:100 | Mouse monoclonal | Proteintech Group, China |
| LACTB | 1:1000 | 1:100 | 1:100 | Rabbit Polyclonal | Proteintech Group, China |
| Ki-67 | / | 1:500 | / | Rabbit Polyclonal | Proteintech Group, China |
